# Utilization of Fe-Ethylenediamine-N,N′-Disuccinic Acid Complex for Electrochemical Co-Catalytic Activation of Peroxymonosulfate under Neutral Initial pH Conditions

**DOI:** 10.3390/molecules28176290

**Published:** 2023-08-28

**Authors:** Bolin Zhang, Yu Chen, Yongjian Wang, Igor Ying Zhang, Rongfu Huang

**Affiliations:** 1 Sichuan Provincial Key Laboratory of Universities on Environmental Science and Engineering, MOE Key Laboratory of Deep Earth Science and Engineering, College of Architecture and Environment, Sichuan University, Chengdu 610065, China; 2 Shanghai Key Laboratory of Molecular Catalysis and Innovation Materials, Collaborative Innovation Centre of Chemistry for Energy Materials, MOE Laboratory for Computational Physical Science, Shanghai Key Laboratory of Bioactive Small Molecules, Department of Chemistry, Fudan University, Shanghai 200433, China

**Keywords:** naphthenic acids, petroleum wastewater, ethylenediamine-N,N′-disuccinic acid, electrochemical catalysis

## Abstract

The ethylenediamine-N,N′-disuccinic acid (EDDS) was utilized to form Fe-EDDS complex to activate peroxymonosulfate (PMS) in the electrochemical (EC) co-catalytic system for effective oxidation of naphthenic acids (NAs) under neutral pH conditions. 1-adamantanecarboxylic acid (ACA) was used as a model compound to represent NAs, which are persistent pollutants that are abundantly present in oil and gas field wastewater. The ACA degradation rate was significantly enhanced in the EC/PMS/Fe(III)-EDDS system (96.6%) compared to that of the EC/PMS/Fe(III) system (65.4%). The addition of EDDS led to the formation of a stable complex of Fe-EDDS under neutral pH conditions, which effectively promoted the redox cycle of Fe(III)-EDDS/Fe(II)-EDDS to activate PMS to generate oxidative species for ACA degradation. The results of quenching and chemical probe experiments, as well as electron paramagnetic resonance (EPR) analysis, identified significant contributions of ^•^OH, ^1^O_2_, and SO_4_^•−^ in the removal of ACA. The ACA degradation pathways were revealed based on the results of high resolution mass spectrometry analysis and calculation of the Fukui index. The presence of anions, such as NO_3_^−^, Cl^−^, and HCO_3_^−^, as well as humic acids, induced nonsignificant influence on the ACA degradation, indicating the robustness of the current system for applications in authentic scenarios. Overall results indicated the EC/PMS/Fe(III)-EDDS system is a promising strategy for the practical treatment of NAs in oil and gas field wastewater.

## 1. Introduction

Bitumen, or oil sand, is an important type of unconventional petroleum reservation. Approximately 110 million tons of bitumen are consumed annually worldwide [1]. The bitumen production process generates large amounts of oil sand process water (OSPW), which is a complex mixture containing naphthenic acids (NAs), ammonia, sulphate, chloride, aromatic hydrocarbons, and trace metals [2]. The NAs are primary persistent organic pollutants that are abundantly present in OSPW and that have a known toxicity. For example, exposure to NAs has been reported to result in abnormal expression of pro-inflammatory cytokine genes in the gills, kidney, and spleen of goldfish [3]. OSPW also presents a major ecotoxicological risk for microalgae [4,5]. Therefore, it is urgently necessary to develop efficient approaches for treatment of NAs.

In recent years, various treatment strategies targeting NAs have been widely studied, with reported advantages and disadvantages. For instance, ozonation of NAs has been demonstrated as an effective method for the degradation of NAs, but it has a high cost [6]. The classical Fenton process has also been studied for efficient removal of NAs, but it comes with the inevitable production of iron sludge and the requirement of acidic initial pH conditions. Due to that, the hydrolysis of Fe and self-decomposition of H_2_O_2_ may occur in the neutral or basic conditions, reducing the oxidation efficiency of the system [7]. Compared with the oxidant H_2_O_2_ in the Fenton reaction, PMS is a new type of oxidant being widely used in water treatment due to its high oxidation capacity and high stability for long-term storage [8]. Various PMS activation methods have been developed to enhance its efficiency and applicability, such as base/PMS, UV/PMS, heat/PMS, transition metal ions/PMS, and biochar/iron oxide composite/PMS [5,9,10,11,12]. Among these methods, the EC/PMS/Fe(III) system has been reported for efficient removal of NAs. It has merits, but it requires the initial pH to be adjusted to the acidic condition [13].

To solve the issue of pH restriction, different complexing agents have been applied to form stable complexes with Fe to prevent its hydrolysis in neutral or basic conditions, including ethylenediaminetetraacetic acid (EDTA), nitrilotriacetic acid (NTA), and ethylenediamine-N,N’-disuccinic acid (EDDS) [14]. Among these complexing agents, EDDS has gained much attention due to its high biodegradability, high stability, and lower toxicity to plants, fungi, and other microorganisms [15]. In fact, EDDS has been reported to have similar complexing capacity to EDTA, with higher biodegradability than EDTA and NTA [16]. It was reported that EDDS-modified Fenton reactions were more effective in the degradation of cyclohexanoic acid (CHA) than classical Fenton process under neutral pH conditions [17]. Furthermore, it was also reported that EDDS could be applied to improve the efficiency of Fe(III)-EDDS-modified electro-Fenton reactions for the degradation of aromatic pollutants like the butylated hydroxyanisole (BHA) at neutral pH conditions [18].

In this work, we reported the application of Fe-EDDS complex to activate PMS in the electrochemical co-catalytic system in order to generate oxidative species to degrade NAs under neutral initial pH conditions. The NAs are composed mainly carboxylic acids, with a general formula of C_n_H_2n+z_O_x_, where n denotes the number of carbon atoms, z indicates the hydrogen deficiency resulting from the formation of ring and/or double bonds in the molecule, and x specifies the number of oxygen atoms in the molecule [19,20]. Due to its complexity, 1-adamantanecarboxylic acid (ACA) has been selected as a model compound to represent NAs for the investigation of the oxidation mechanisms in previous studies of the treatment of NAs, and it was thus used as the model compound in this study [11,21]. The degradation mechanisms of the current EC/PMS/Fe(III)-EDDS process has been investigated in several aspects: (i) to compare various systems and to explore different influencing factors on the degradation rate of ACA; (ii) to clarify the contribution of different oxidative species generated in the system for ACA removal; (iii) to explore the pathways of ACA degradation and to assess the toxicity of ACA byproducts; and (iv) to examine the impact of nitrate ions, chloride ions, bicarbonate ions, and humic acids on the ACA removal rate.

## 2. Results and Discussion

### 2.1. Comparison of ACA Degradation in Different Systems

**Figure 1:**
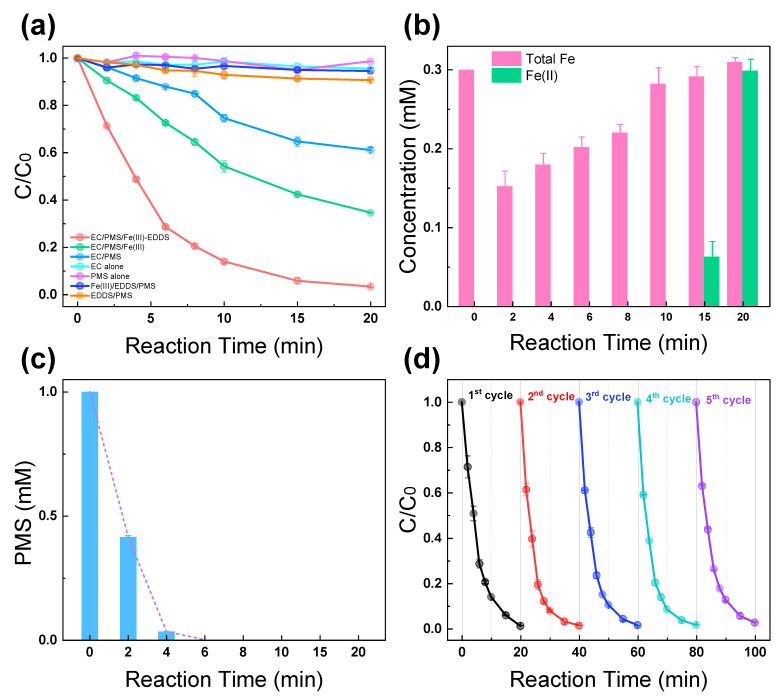
(**a**) Degradation of ACA in different systems. (**b**) The concentrations of Fe(II) and total Fe, and (**c**) the PMS consumption in the EC/PMS/Fe(III)-EDDS system. (**d**) The cycle use of electrodes in five repeated processes. Reaction conditions: [ACA] = 0.1 mM, [PMS] = 1 mM, [EDDS] = 0.6 mM, [Fe(III)] = 0.3 mM, [Na_2_SO_4_] = 20 mM, current density = 8.163 mA/cm^2^, and initial pH = 7.0.

Figure 1a illustrates the degradation curves of ACA in various systems, namely EC/PMS/Fe(III)-EDDS, EC/PMS/Fe(III), EC/PMS, EC alone, PMS alone, PMS/Fe(III)-EDDS, and EDDS/PMS under neutral initial pH conditions. A slight degradation rate (<10%) was observed in the EC alone, PMS alone, Fe(III)-EDDS/PMS, and EDDS/PMS processes within a 20 min reaction time, indicating that EC alone and PMS alone processes are not effective in ACA removal, and PMS could not be efficiently activated by EDDS or Fe(III)-EDDS in order to generate reactive species to remove ACA. The slight removal rate in these PMS-containing processes may be because of the PMS self-decomposition at pH 7 [22]. The ACA degradation rate in the EC/PMS process was 38.5%, probably attributed to the direct activation of PMS by the electric current, as indicated by Equations (1) and (2) [23].

(1) $${HSO}_{5}^{-}+e^{-}\to {SO}_{4}^{•-}+{OH}^{-}$$

(2) HSO5−+e−→SO42−+OH•

The ACA removal rate exhibited a substantial increase in the EC/PMS/Fe(III)-EDDS system (96.6%) compared to that of the EC/PMS/Fe(III) system (66.7%), with the Faraday efficiency of the former system being 15.7%. This improvement was attributed to the enhanced activation of PMS through the Fe(III)/Fe(II) redox cycle triggered under EC conditions. Furthermore, the addition of EDDS facilitated the formation of a stable Fe-EDDS complex, preventing Fe hydrolysis under neutral pH conditions. The above results reveal that an electric current and the redox cycle of Fe(III)-EDDS/Fe(II)-EDDS could work in synergy to efficiently activate PMS, leading to complete degradation of ACA in the system. In addition, compared with the previous studies about NA removal, this system also achieved comparable results, as shown in Table S1. Overall, the addition of EDDS greatly promoted the activation of PMS under neutral pH conditions in the EC/PMS/Fe(III)-EDDS system in order to generate oxidative species to effectively remove ACA.

Figure 1b illustrates the trends of concentration of Fe(II) and total Fe in the EC/PMS/Fe(III)-EDDS system during a 20 min reaction time. In this process, the concentration of total Fe dropped rapidly in the first 2 min, likely due to the adsorption of the carbon felt cathode that has a high specific surface. The concentration of total Fe indicates the total Fe in the solution. This is consistent with the result in a previous study [24]. After 2 min, the concentration of total Fe gradually increased, perhaps due to the fact that the adsorbed Fe was gradually dissolved with the assistance of the complexing agent EDDS. Fe(II) was not detected in the beginning of the reaction; Fe(II) was completely consumed to activate PMS. Accumulation of Fe(II) was detected after 15 min, likely because the PMS was completely consumed at that time (Figure 1c). PMS was consumed at a faster rate in the current system than that in the previous study [25,26], indicating the effectiveness of the redox cycle of Fe(III)-EDDS/Fe(II)-EDDS for PMS activation. Additionally, an FT-IR analysis was conducted to investigate the chemical stability of the iron complex. As shown in Figure S1, the characteristic peaks of Fe(III)-EDDS before and after the electrochemical oxidation process remained similar, indicating that the Fe(III)-EDDS remained stable throughout the reaction process.

Figure 1d illustrates the degradation curves of five consecutive processes in the EC/PMS/Fe(III)-EDDS system, in order to evaluate the reusability of the system. The electrodes were not rinsed between processes. It was observed that the removal rate of ACA remained as high as 97.4% in the fifth cycle, indicating the excellent reusability and stability of current system. In addition, Figure S2 shows the SEM images of the carbon felt cathode after the treatment of the EC/Fe(III)-EDDS/PMS and EC/Fe(III)/PMS systems. It was observed that relatively more iron precipitates were found in Figure S2b, likely due to the hydrolysis of iron ions under neutral pH conditions. The addition of EDDS led to the formation of iron-EDDS complex that prevented the hydrolysis of iron ions and maintained the carbon felt cathode in a clean condition, resulting in the enhancement of the reusability and stability of the system with a practical significance.

**Figure 2:**
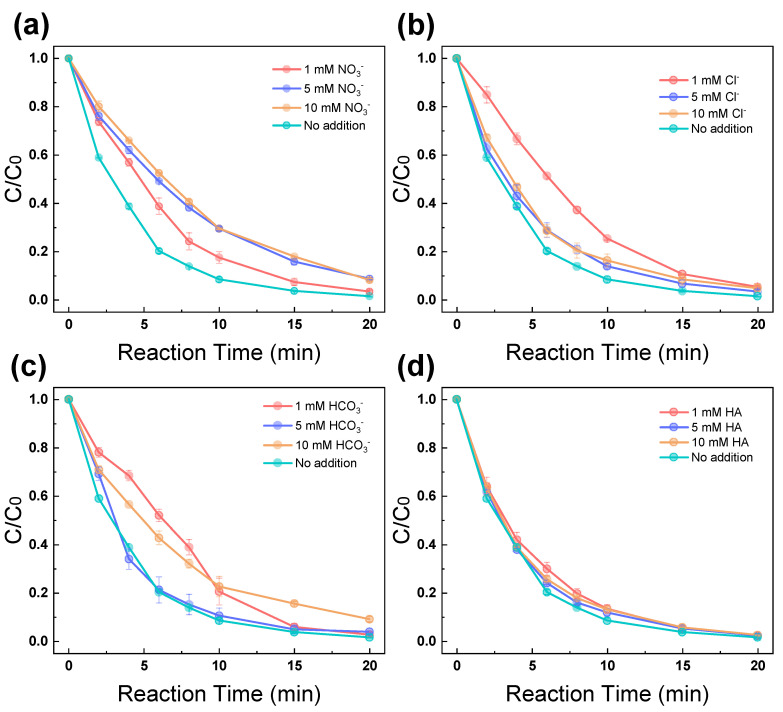
Effect of (**a**) NO_3_^−^, (**b**) Cl^−^, (**c**) HCO_3_^−^, and (**d**) HA on the degradation of ACA in the EC/PMS/Fe(III)-EDDS system. Reaction conditions: [ACA] = 0.1 mM, [PMS] = 1 mM, [EDDS] = 0.6 mM, [Fe(III)] = 0.3 mM, [Na_2_SO_4_] = 20 mM, current density = 8.163 mA/cm^2^, and initial pH = 7.0.

In a natural water background, various types of anions, as well as humic acid (HA), are commonly present, which may function as radical scavengers to reduce the oxidation performance of the system. In this study, three anions and an organic acid were examined, including NO_3_^−^, Cl^−^, HCO_3_^−^, and HA. As shown in Figure 2a, upon the addition of different concentrations NO_3_^−^ to the reaction system, a minor inhibition was observed for the degradation efficiency of ACA, indicating NO_3_^−^ may induce a negative impact to the removal of ACA, likely due to its reaction with SO_4_^•−^ to quench a portion of radicals [27]. Figure 2b shows the ACA degradation curves under the influence of different dosages of Cl^−^. The addition of 1 mM Cl^−^ resulted in a minor reduction in the removal rate, from 86.1% to 74.6%, within 10 min. This is likely because Cl^−^ could scavenge the radicals in the reaction system and lead to the consumption of ^•^OH radicals, as reported in previous studies [23,28]. Remarkably, as the dosage of Cl^−^ increased to 5 mM or 10 mM, the ACA degradation rate demonstrated a notable improvement in comparison to the rate observed at a Cl^−^ dosage of 1 mM. This is likely because a considerable amount of Cl^•^ may be produced from the excessive Cl^−^ under electrochemical conditions, which contribute to the oxidation system for ACA removal [29].

Figure 2c shows the ACA degradation curves under the influence of different dosages of HCO_3_^−^. A minor inhibition was observed for the degradation efficiency of ACA, indicating that HCO_3_^−^ induced negative influence on the removal of ACA due to its reaction with free radicals in the system [30]. Figure 2d shows the ACA degradation curves under the influence of different dosages of HA, which is naturally present in different water bodies. It was observed that the addition of HA exerted a nonsignificant influence on the ACA removal of the system. To sum up, the presence of NO_3_^−^, Cl^−^, HCO_3_^−^, and HA exerted a nonsignificant influence on the EC/PMS/Fe(III)-EDDS process for ACA removal, indicating that the current system is robust enough to be applied for future practical treatment of oil and gas field wastewater containing background anions.

### 2.2. Oxidation Mechanisms of the EC/PMS/Fe(III)-EDDS System

**Figure 3:**
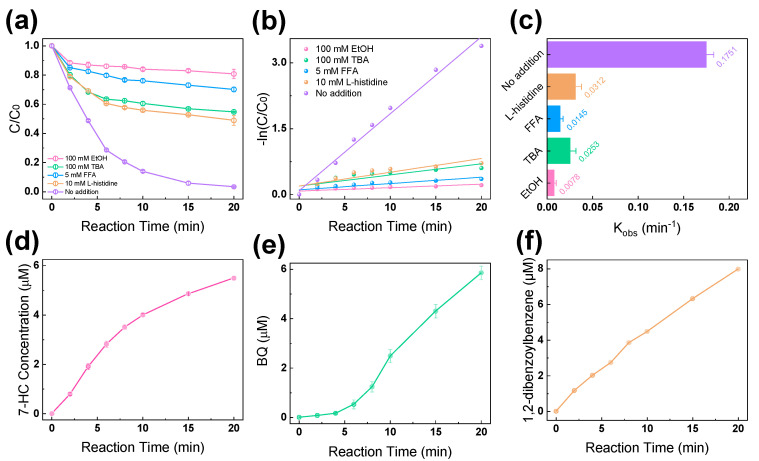
(**a**) Effects of different quenchers on the degradation of ACA in the EC/PMS/Fe(III)-EDDS system. (**b**) Pseudo-first-order plots of the degradation of ACA with different quenchers. (**c**) The reaction kinetics for ACA treatment with different quenchers. (**d**) The production of 7-hydroxycoumarin (7-HC) indicated the generation of ^•^OH in the EC/PMS/Fe(III)-EDDS system. (**e**) The chemical probe p-hydroxybenzoic acid (p-HBA) could react with SO_4_^•−^ to generate benzoquinone (BQ) in the system. (**f**) The production of 1,2-dibenzoylbenzene indicated the presence of ^1^O_2_ in the system. Reaction conditions: [ACA] = 0.1 mM, [PMS] = 1 mM, [EDDS] = 0.6 mM, [Fe(III)] = 0.3 mM, [Na_2_SO_4_] = 20 mM, [p-HBA] = 1.8 mM, [coumarin] = 2 mM, [DPBF] = 0.1 mM, current density = 8.163 mA/cm^2^, and initial pH = 7.0.

Figure 3a compares the ACA degradation curves of the EC/PMS/Fe(III)-EDDS system in the presence of different scavengers, such as tert-butanol (TBA), ethanol, furfuryl alcohol (FFA), and L-histidine, to evaluate the contribution of different oxidative species to the system. Figure 3b,c show the calculation process and the results of the reaction rate constants of the ACA degradation processes in the presence of different scavengers. It is known that tert-butanol (TBA) is an effective quencher of ^•^OH, with a high rate constant (*k*(^•^OH) = 6.0 × 10^8^ M^−1^s^−1^) [31]. It was observed that, with the addition of 100 mM TBA, the ACA degradation rate reduced from 96.6% to 45.2%, indicating that the ^•^OH played a significant role in the degradation of ACA. Both SO_4_^•−^ and ^•^OH could be scavenged by ethanol (EtOH), with rate constants of *k*(^•^OH) = 1.9 × 10^9^ M^−1^s^−1^ and *k*(SO_4_^•−^) = 1.6 × 10^7^ M^−1^s^−1^ [31]. After adding 100 mM EtOH, the removal rate of ACA was reduced from 96.6% to 19.2%, and the *k*_obs_ value was reduced from 0.1751 to 0.0078, indicating the presence of SO_4_^•−^ in the system in addition to ^•^OH. The furfuryl alcohol (FFA) was selected to quench ^1^O_2_, as it can react with ^1^O_2_ at a relatively high rate constant of 1.2 × 10^8^ M^−1^s^−1^. Given that the FFA can also scavenge ^•^OH, only 5 mM FFA was added into the system to ensure that it would scavenge a similar amount of ^•^OH with TBA based on the ratio of their rate constants [32]. It was observed that the addition of FFA decreased the ACA removal rate to 29.9%, which is apparently lower than that of the TBA-involving process, indicating that ^1^O_2_ was generated in the process to contribute to ACA removal. Meanwhile, the L-histidine was also used to scavenge ^1^O_2_, and it induced a reduction in ACA removal from 96.6% to 51.1%, corroborating that the ^1^O_2_ has made a considerable contribution in the oxidation system.

A chemical probe experiment was conducted in order to analyze the oxidative radicals generated in the degradation process. The coumarin was usually employed as a chemical probe to react with ^•^OH to produce 7-hydroxycoumarin (7-HC) [33]. As shown in Figure 3d, the concentration of 7-HC increased rapidly to reach 5.489 μM at 20 min reaction time, indicating the generation of ^•^OH in the system. The p-hydroxybenzoic acid (p-HBA) was usually employed as a chemical probe to react with SO_4_**^•^**^−^ to produce p-benzoquinone (p-BQ) [34]. As depicted in Figure 3e, p-BQ gradually increased to 5.854 μM within a 20 min reaction time, indicating the generation of SO_4_**^•^**^−^ in the system. In addition, 1,3-diphenylisobenzofuran (DPBF) was utilized as the chemical probe to react with the ^1^O_2_ to produce 1,2-dibenzoylbenzene in the system. As shown in Figure 3f, the concentration of 1,2-dibenzoylbenzene increased to 7.979 μM within 20 min, implying the generation of ^1^O_2_ in the EC/PMS/Fe(III)-EDDS system. In short, the ACA removal in the EC/PMS/Fe(III)-EDDS system appears to be the collaborative contribution of different oxidative species, including OH, ^1^O_2_, and SO_4_^•−^.

**Figure 4:**
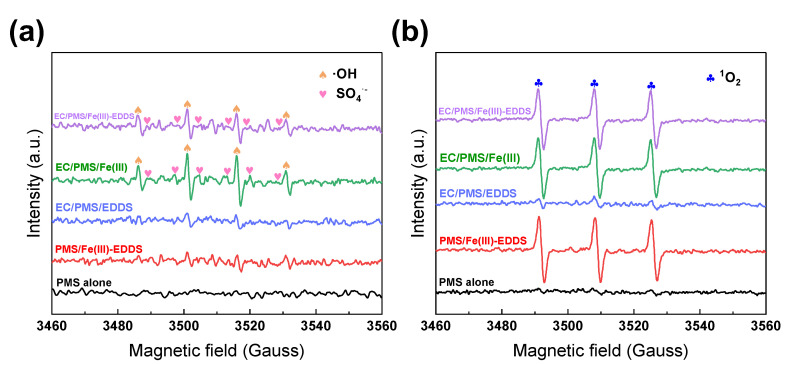
The electron paramagnetic resonance spectroscopy of (**a**) DMPO-^•^OH, DMPO-SO_4_**^•−^**, and (**b**) TEMP-^1^O_2_ in different systems. Reaction conditions: [ACA] = 0.1 mM, [PMS] = 1 mM, [EDDS] = 0.6 mM, [Fe(III)] = 0.3 mM, [Na_2_SO_4_] = 20 mM, current density = 8.163 mA/cm^2^, and initial pH = 7.0.

EPR analysis is typically applied as an established technique for the direct qualitative analysis of radicals [35]. The EPR analysis was conducted in the PMS alone, PMS/Fe(III)-EDDS, EC/PMS/EDDS, EC/PMS/Fe(III), and EC/PMS/Fe(III)-EDDS systems using DMPO (5,5-dimethyl-1-pyrrolidine-N-oxide) and TEMP (2,2,6,6-tetramethyl-4-piperidinyloxyl) as radical spin-trapping reagents. These reagents were able to trap free radicals to form DMPO-^•^OH, DMPO-SO_4_**^•^**^−^ and TEMP-^1^O_2_ adducts, to be detected in EPR analysis [36]. As shown in Figure 4a, no signal peak of DMPO-^•^OH or DMPO-SO_4_**^•−^** was observed in the PMS alone, PMS/Fe(III)-EDDS, and EC/PMS/EDDS systems. However, strong peaks (peak height ratio of 1:2:2:1) of DMPO-^•^OH and DMPO-SO_4_**^•−^** were observed in the EC/PMS/Fe(III) and EC/PMS/Fe(III)-EDDS systems, indicating that both ^•^OH and SO_4_**^•−^** were present in the two systems.

To further investigate the contribution of ^1^O_2_, TEMP was used to detect its presence in five processes, and the results are displayed in Figure 4b. It was observed that no peak signal was detected in the PMS alone system, indicating that PMS self-decomposition was not sufficient in the PMS alone system to produce ^1^O_2_. The EC/PMS/EDDS system exhibited no peak detection, indicating the crucial role of Fe(III) in activating PMS to generate ^1^O_2_. Notably, the signal peaks in the other three processes displayed detectable intensity. These results indicated that ^1^O_2_ was generated in three processes, and Fe(III) played a more important role than EC and EDDS.

**Figure 5:**
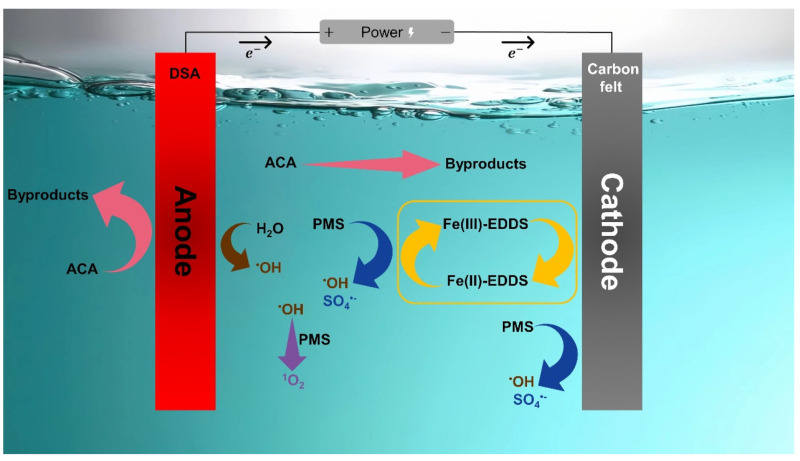
Illustration of the oxidation mechanisms for ACA removal in the EC/PMS/Fe(III)-EDDS process.

In summary, based on the above discussion, it can be confirmed that ^•^OH, SO_4_**^•−^**, and ^1^O_2_ are major contributors to the removal process of ACA in the EC/PMS/Fe(III)-EDDS system, as per Equations (3)–(5). These radicals are mainly produced from PMS activation [37]. Figure 5 illustrates the multiple routes of oxidation mechanisms obtained through the above experimental results.

(3) $${HSO}_{5}^{-}+Fe\left(II\right)EDDS\to {SO}_{4}^{•-}+{OH}^{-}+Fe\left(III\right)EDDS$$

(4) HSO5−+FeIIEDDS→SO42−+OH•+FeIIIEDDS

(5) HSO5−+SO52−+OH•+e−→2SO42−+O12+H2O

### 2.3. ACA Degradation Pathways and Toxicity Assessment of Degradation Byproducts

The UHPLC-QTOF-MS analysis was, firstly, performed to obtain the chemical formulae of ACA byproducts based on their accurate masses and the match of isotopic patterns on the mass spectra. Then, the chemical structures of ACA byproducts were derived according to their chemical formulae and the predicted reactive sites based on the calculation of Fukui index. Toxicity assessment was performed using the Toxicity Estimation Software Tool (T.E.S.T., v17.0.2.0) based on the chemical structures of ACA and ACA byproducts.

**Figure 6:**
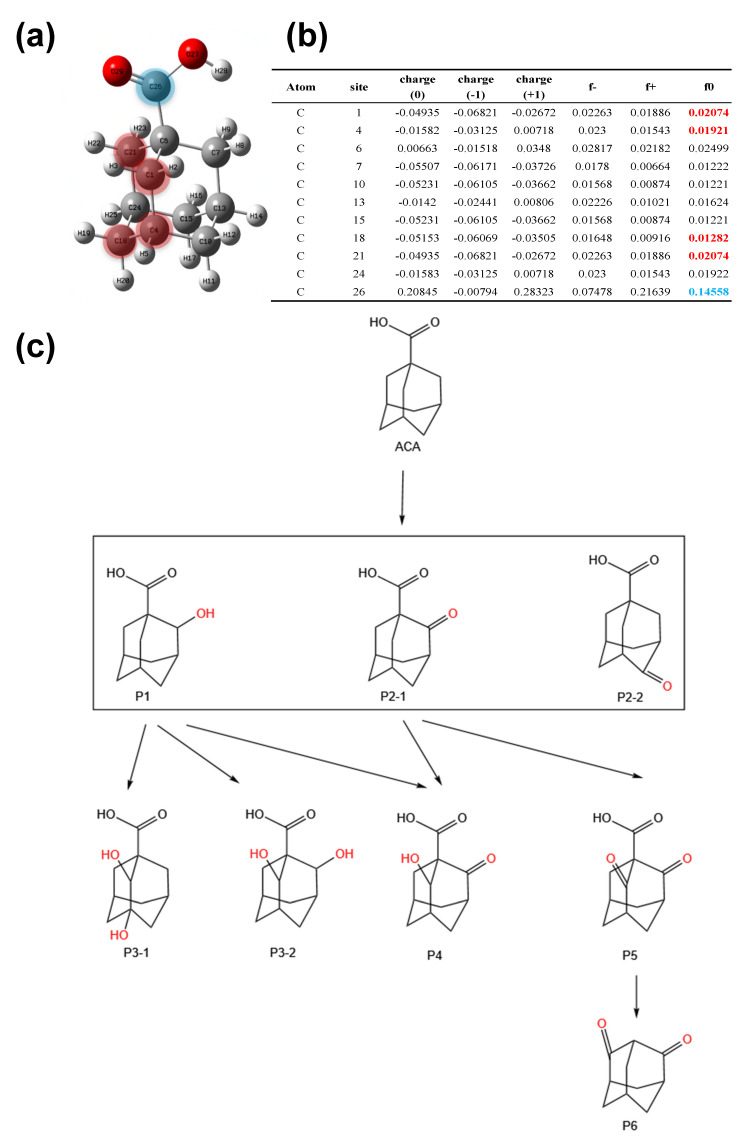
(**a**) The structure of ACA molecule. (**b**) Calculated Fukui indexes of different reactive sites on the ACA molecule. (**c**) The degradation pathways of ACA degradation in the EC/PMS/Fe(III)-EDDS system.

Figure 6 illustrates the chemical structure of ACA, along with the calculated Fukui indexes corresponding to its reaction sites in the EC/PMS/Fe(III)-EDDS system. Furthermore, it presents the degradation pathways of ACA within the mentioned system. The Fukui index was calculated to reveal the reactive sites of ACA in order to predict its degradation pathways and byproducts [38]. The basis set 6-311+G (3df, 2p) was typically selected for theoretical calculation due to its superiority in kinetics, based on free radical reactions and modeling [39]. The calculation process of the Fukui indexes was summarized in Text S1. Table S2 summarizes the information of eight ACA degradation byproducts identified using UHPLC-QTOF-MS analysis. Previous studies on ACA degradation reported that ACA generated hydroxyl-substituted, carbonyl-substituted, and decarboxylated byproducts in different degradation pathways [11,21]. As shown in Figure 6b, the Fukui function revealed that C1, C4, C18, and C21 had relatively high f0 values that were labeled red, indicating that these sites are relatively more susceptible to free radical attack rather than other sites. In Figure 6c, site C1 was attacked by ROS to form hydroxyl-ACA (P1), which then underwent further attack to turn into the byproducts P3-1, P3-2, and P4. In addition, carbonyl-ACA (P2-1 and P2-2) could be generated when the C21 or C18 of ACA was replaced by a carbonyl group. P2-1 could be attacked to form the byproduct with two carbonyl groups (P5), which could further lose the carboxyl group on C26 to produce the decarboxylated byproduct (P6).

Figure S3 shows the evolving trends of the relative abundances of eight ACA byproducts during a 20 min reaction time, and it can be observed that these byproducts initially increased and then decreased over time, indicating these byproducts were initially generated from ACA degradation and then further degraded into small molecules or mineralized. Among all ACA byproducts, P1, P2-1, and P2-2 were the main byproducts, due to their high relative abundances. Figure S4 shows trends of TOC variation of the sample in the EC/PMS/Fe(III)-EDDS process within 60 min. A TOC removal of about 40% was observed at a reaction time of 60 min, indicating that the mineralization of ACA and its byproducts could be achieved in the EC/PMS/Fe(III)-EDDS system with an extended reaction time.

**Figure 7:**
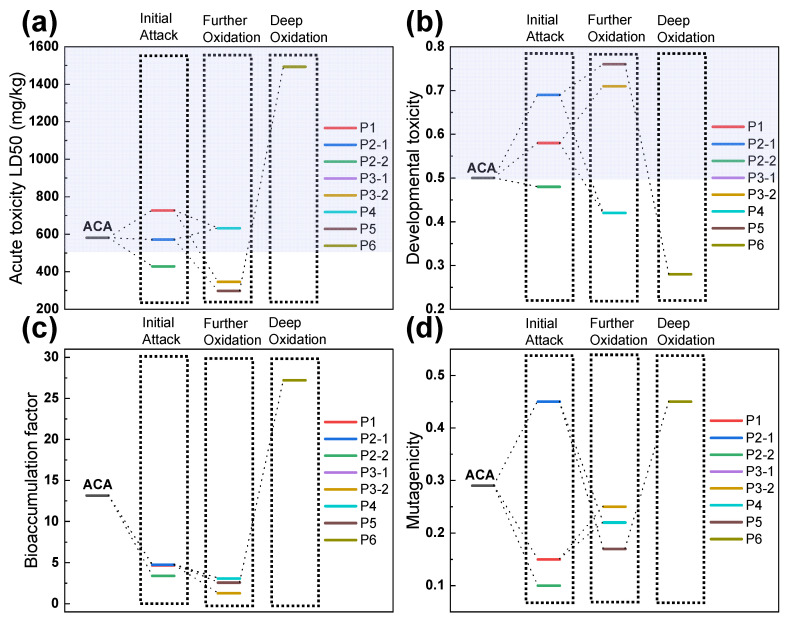
The toxicity results of ACA and 8 byproducts generated in the EC/PMS/Fe(III)-EDDS system, in terms of the (**a**) acute toxicity, (**b**) developmental toxicity, (**c**) bioaccumulation factor, and (**d**) mutagenicity. These toxicity results were obtained from calculations using the Toxicity Estimation Software Tool (T.E.S.T.).

In order to assess the environmental impact of these byproducts further, toxicological analysis was performed based on the chemical structure of the ACA byproducts. Figure 7 shows the toxicity results of ACA and its byproducts, including acute toxicity, bioaccumulation factor, developmental toxicity, and mutagenicity, obtained using the Toxicity Estimation Software Tool (T.E.S.T.) based on the quantitative structure–activity relationship (QSAR) model [40,41,42]. Figure 7a presents a clear and significant reduction in the acute toxicity LD50 values of P2-2, P3-1, P3-2, and P5 compared to ACA (LD50 of 581.33 mg/kg). This indicates a notable increase in the acute toxicity of these byproducts relative to ACA. Despite their heightened structural acute toxicity, all of these byproducts were efficiently eliminated within just 20 min of reaction time, as illustrated in Figure S3. The developmental toxicity of the byproducts was either lower (P2-2, P4, and P6) or higher (P1, P2-1, P3-1, P3-2, and P5) than that of ACA (Figure 7b). Figure 7c shows that the bioaccumulation factor of all byproducts decreased to a certain degree, except for P6. Furthermore, P2-1 and P6 induced increased mutagenicity compared to ACA (Figure 7d). Based on above results, despite the fact that some of the ACA byproducts may induce increased toxicity, these ACA byproducts could be mineralized completely with extended treatment time in the EC/PMS/Fe(III)-EDDS system in order to eventually eliminate their toxicity.

### 2.4. Degradation of Fluka Commercial NA Mixture

The Fluka commercial NA mixture is composed of a complex class of NAs extracted from crude oil, and it has been extensively employed as a representative of organic pollutants in authentic oil and gas field wastewater samples in previous studies [37,43]. The composition of the Fluka NA mixture predominantly features classical NAs, characterized by a double-bond equivalent (DBE) number that spans the range of 0 to 6, and a carbon number that spans the range of 7 to 26. In this work, the degradation of the Fluka commercial NA mixture standard has been examined in the EC/PMS/Fe(III)-EDDS process under neutral pH conditions in order to evaluate the applicability of the current system for the practical treatment of authentic wastewater.

**Figure 8:**
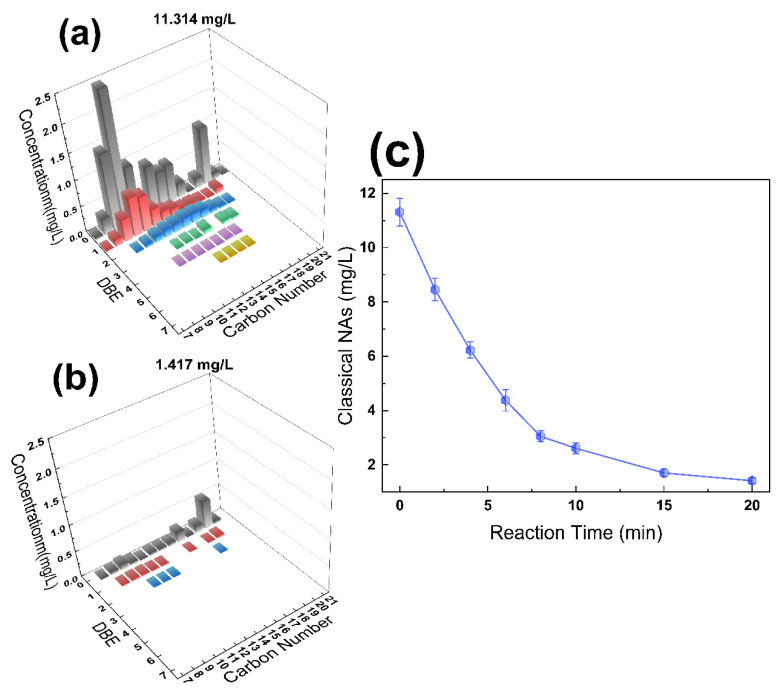
The distributions of classical NAs, in terms of carbon number and double-bond equivalent (DBE) number, before (**a**) and after (**b**) treatment in the EC/PMS/Fe(III)-EDDS system. Different colors represent different DBE numbers that indicate the hydrogen deficiency in an organic molecule. (**c**) The degradation curve of classical NAs in the EC/PMS/Fe(III)-EDDS system is shown within a 20 min reaction time.

Figure 8a,b present the distributions of NAs in the sample, categorized by carbon and DBE numbers, both prior to and after treatment in the EC/PMS/Fe(III)-EDDS system under neutral initial pH conditions. The treatment yielded a substantial reduction in NA concentration, from 13.668 mg/L to 1.417 mg/L, along with a decline in the number of distinct NA species, from 56 to 25. Detailed data can be found in Tables S3 and S4. Additionally, Figure 8c illustrates the removal curve of ACA, treated by the EC/PMS/Fe(III)-EDDS process within a 20 min reaction time, demonstrating an 89.6% removal of classical NAs during this period, with complete removal expected during prolonged treatment. To conclude, the EC/PMS/Fe(III)-EDDS process exhibits significant potential for the realistic treatment of OSPW in the future.

## 3. Materials and Methods

### 3.1. Chemicals

The chemicals used in this work, including ACA, are listed in Table S5 of Supplementary Materials. The chemicals were utilized as received without applying additional purification processes. A Milli-Q system was utilized to prepare deionized water (>18.2 MΩ cm^−1^).

### 3.2. Degradation Experiment

An initial volume of 150 mL of the sample with 0.1 mM ACA as the model pollutant and 20 mM NaSO_4_ as the electrolyte was added to a glass reactor (6 cm I.D. × 9 cm height). A total of 0.6 mM EDDS and 0.3 mM Fe(III) were subsequently added with the concentration ratio of 2:1 [37]. The solution was uniformly mixed using a magnetic stir bar, which continuously stirred throughout the entire reaction process to ensure efficient mass transfer within the system. The initial pH of the sample was adjusted to 7.0 ± 0.1 by dropwise addition of 0.1 mM H_2_SO_4_ or NaOH at room temperature (23 °C) before adding 1.0 mM PMS. The anode was a 35 mm × 35 mm × 1 mm Ti/RuO_2_-IrO_2_ plate and the cathode was 35 mm × 35 mm × 3 mm carbon felt. In the reactor setup, the anode and cathode were placed 18 mm apart, and 100 mA constant current was applied using a direct-current power source (APS3003S–3D, GRATTEN, Nanjing, China). Sample analysis involved extracting 500 μL from the reactor at specific time points, which was filtered using a 0.22 μm PTFE membrane and then injected into HPLC vials. These vials were prefilled with 500 μL of methanol, for the purpose of scavenging reactive radicals in the sample. All experimental results presented in this study are the average values calculated from duplicate samples, with corresponding error bars to represent the standard deviations.

### 3.3. Analytical Methods

The ultra-high-performance liquid chromatography quadrupole time-of-flight mass spectrometry (UHPLC-QTOF-MS, G6545, Agilent, Santa Clara, CA, USA) with an electrospray ionization (ESI) source was utilized for comprehensive characterization of ACA, ACA degradation byproducts, and the commercial NA mixture. Detailed information can be found in Texts S2–S4. To characterize three types of free radicals, we employed electron paramagnetic resonance (EPR) analysis (Bruker EMX plus X-band CW EPR spectrometer, Billerica, MA, USA; microwave frequency, 9.83 GHz; microwave power, 2.00 MW). The HPLC (Prominence RF-20A, Shimadzu, Tokyo, Japan) was employed for measuring the free radical probes of ^1^O_2_, ^•^OH, and SO_4_^•−^, with additional information available in Texts S5–S7. For the determination of Fe(II), total Fe, and PMS concentrations, we used the UV-vis spectrophotometer (UV-3600, Shimadzu, Japan), and the methodology is detailed in Text S8. Additionally, the total organic carbon (TOC) of the samples was analyzed using the TOC analyzer (TOC-L CPH, Shimadzu, Japan).

## 4. Conclusions

This study investigated the oxidation mechanisms and degradation pathways of the EC/PMS/Fe(III)-EDDS system for the degradation of ACA under neutral pH conditions. The addition of EDDS improved the stability of Fe(III) and Fe(II) at pH 7 to prevent the hydrolysis of iron ions and thus promoted the redox cycle of Fe(III)-EDDS/Fe(II)-EDDS to activate PMS in order to generate oxidative species for ACA removal. The SEM images demonstrated that the presence of EDDS could maintain the system in a relatively clean state for obtaining extended reusability. Although the removal rate of ACA was affected slightly in the presence of NO_3_^−^, Cl^−^, HCO_3_^−^, and HA, a desired effect was still achieved, demonstrating the superiority and robustness of this system. The results of the quenching experiment, the chemical probe tests, and the EPR analysis indicated that ^•^OH, SO_4_**^•^**^−^, and ^1^O_2_ are major contributors to the oxidation process. Additionally, the results of high resolution mass spectrometry analysis and Fukui function calculations identified hydroxyl-substituted, carbonyl-substituted, and decarboxylated byproducts resulting from the oxidation of ACA. The degradation pathways of ACA were also derived. Overall, this system effectively removed ACA, as well as a commercial NA mixture, indicating its capacity for the degradation of NAs in future treatment of oil and gas field wastewater, with high efficiency and reliability.

## Data Availability

Data will be provided upon request.

## Supplementary Materials

The following supporting information can be downloaded at: https://www.mdpi.com/article/10.3390/molecules28176290/s1, Figure S1: FT-IR patterns of Fe(III)-EDDS before (a) and after (b) reaction; Figure S2: Scanning Electron Microscope (SEM) images of the carbon felt cathode after the treatment of EC/Fe(III)-EDDS/PMS (a) and EC/Fe(III)/PMS (b) systems; Figure S3: The relative abundance trends of all byproducts generated during the EC/PMS/Fe(III)-EDDS system removing ACA; Figure S4: The TOC degradation during the reaction process in the EC/PMS/Fe(III)-EDDS system. Table S1: Comparison with previous paper in the NAs degradation; Table S2: Summary of ACA degradation byproducts; Table S3: The distributions of classical NAs, in terms of carbon number and double-bond equivalent (DBE) number, before treatment in the EC/PMS/Fe(III)-EDDS system; Table S4: The distributions of classical NAs, in terms of carbon number and double-bond equivalent (DBE) number, after treatment in the EC/PMS/Fe(III)-EDDS system; Table S5: Chemicals and Reagents. Text S1: Calculation based on Fukui function; Text S2: UHPLC-QTOF-MS analysis of ACA; Text S3: UHPLC-QTOF-MS analysis of ACA degradation byproducts; Text S4: UHPLC-QTOF-MS analysis of Fluka commercial NAs mixture; Text S5: The analysis of singlet oxygen (^1^O_2_); Text S6: The analysis of hydroxyl radical (•OH); Text S7: The analysis of sulfate radical (SO_4_^•−^); Text S8: The analysis of iron species and PMS. References [44,45,46,47,48,49,50,51,52] are cited in the Supplementary Materials.
