# Supplementary material for: Utilization of Fe-Ethylenediamine-N,N′-Disuccinic Acid Complex for Electrochemical Co-Catalytic Activation of Peroxymonosulfate under Neutral Initial pH Conditions
Source: Molecules. 2023 Aug 28;28(17):6290. doi: 10.3390/molecules28176290 (PMC10488724; doi:10.3390/molecules28176290)
Supplement: Supplementary file 1 [file molecules-28-06290-s001.zip › molecules-2546012-supplementary.pdf]

Supplementary Materials

Utilization of Fe-ethylenediamine-N,N'-disuccinic acid complex for electrochemical activation of peroxymonosulfate under neutral initial pH condition

Bolin Zhang[1], Yu Chen[1], Yongjian Wang[1], Igor Ying Zhang[2,*], Rongfu Huang[1,*]

[1] *Sichuan Provincial Key Laboratory of Universities on Environmental Science and Engineering, MOE Key Laboratory of Deep Earth Science and Engineering, College of Architecture and Environment, Sichuan University, Chengdu 610065, China*

[2] *Shanghai Key Laboratory of Molecular Catalysis and Innovation Materials, Collaborative Innovation Centre of Chemistry for Energy Materials, MOE Laboratory for Computational Physical Science, Shanghai Key Laboratory of Bioactive Small Molecules, Department of Chemistry, Fudan University, Shanghai 200433, China*

Total page: 22

Table: 5

Texts: 8

Figures: 4

* Corresponding authors: Igor Ying Zhang, Ph.D.
Tel./fax: +86 18221165538, Email address: igor_zhangying@fudan.edu.cn
* Corresponding authors: Rongfu Huang, Ph.D.
Tel./fax: +86 19181766064, Email address: rongfu@scu.edu.cn

Table S1. Comparison with previous paper in the NAs degradation

Materials	pH of operation	Efficiency	References
electricity,<br>Fe(III)-EDDS complex,<br>PMS	7.0	96.6%	In this work
biochar/iron oxide (B-FeOx) catalyst,<br>PMS	8.5	91.6%	[1]
UV	8.7	30.9%	[2]
electricity,<br>Fe(III)-NTA complex,<br>PMS	7.0	100.0%	[3]
UV-NTA-Fenton;	7.0	98.4%	[4]
ferric citrate,<br>visible light irradiation	3.0	98.3%	[5]

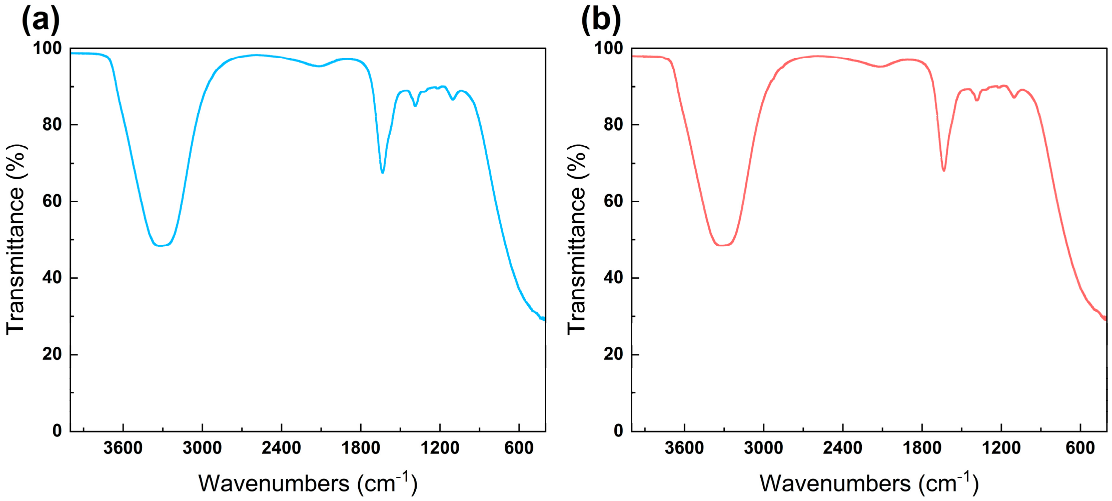

Figure S1. FT-IR patterns of Fe(III)-EDDS before (a) and after (b) reaction.

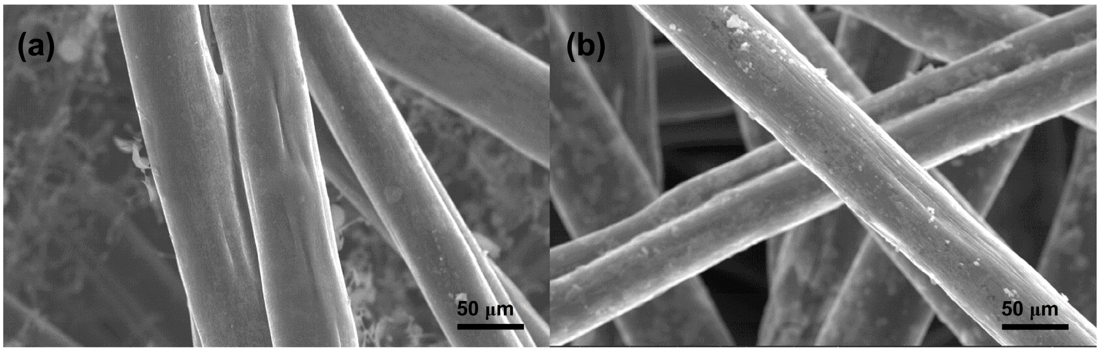

Figure S2. Scanning Electron Microscope (SEM) images of the carbon felt cathode after the treatment of EC/Fe(III)-EDDS/PMS (a) and EC/Fe(III)/PMS (b) systems.

Text S1. Calculation based on Fukui function

First-principle calculations were performed on the electronic-structure package of Gaussian 16 [6]. The state-of-the-art long-range-corrected hybrid meta-GGA method with empirical dispersion, ωB97XD [7], was employed at the basis set level of 6-311+G (3df, 2p). The solvation model based on density (SMD) [8] was employed to take the solvation effect into account. The Multiwfn package [9] was then used to evaluate condensed Fukui functions based on the Hirsh field charge distribution [10].

Fukui function has been widely used for the prediction of reactive sites of electrophilic, nucleophilic, and radical attacking [6,11]. Specifically, Fukui function is defined as:

$$f(r) = \left(\frac{\partial^2 E}{\partial N . \partial v(r)} \right) = \left[\frac{\partial \mu}{\partial v(r)} \right]_N = \left[\frac{\partial \rho(r)}{\partial N} \right]_{V(r)} \quad \text{(S1)}$$

where $\rho(r)$ is the electron density at a point r in space, N is electron number in present system, the constant term v in the partial derivative is external potential. In the condensed version of Fukui function, atomic population number is used to represent the amount of electron density distribution around an atom. The condensed Fukui function can be calculated unambiguously for three situations:

$$\text{Nucleophilic attack: } f_k^+ = q_N^k - q_{N+1}^k \quad \text{(S2)}$$

$$\text{Electrophilic attack: } f_k^- = q_{N-1}^k - q_N^k \quad \text{(S3)}$$

$$\text{Radical attack: } f_k^0 = \left(q_{N-1}^k - q_{N+1}^k \right) / 2 \quad \text{(S4)}$$

where q^k is the atom charge population of atom k at the corresponding state. The Fukui function contains relative information about different sites of one molecule. In this study, radical species were the primary attack species, thus Fukui index for radical

attack was used to analyze the regioselectivity during ACA degradation.

Table S2. Summary of ACA degradation byproducts.

Serial Number	Molecular formula	RT (min)	m/z	Proposed Structure
ACA	C11H16O2	3.369	179.1079	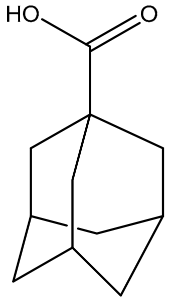
P1	C11H16O3	11.900	195.1029	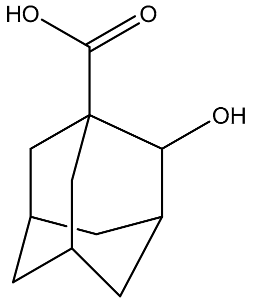
P2-1	C11H14O3	11.748	193.0874	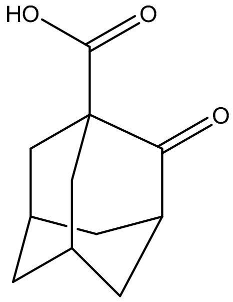
P2-2	C11H14O3	12.280	193.0874	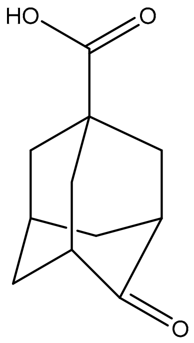

P3-1	C11H16O4	11.116	211.0978	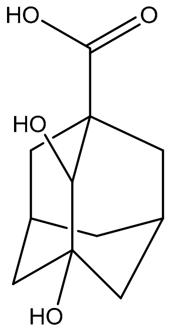
P3-2	C11H16O4	12.280	211.0978	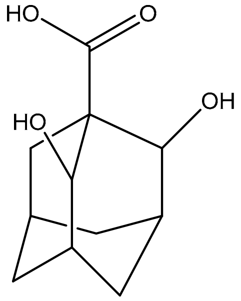
P4	C11H14O4	12.250	209.0821	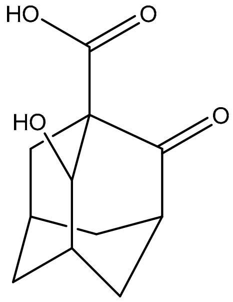
P5	C11H12O4	12.879	207.0662	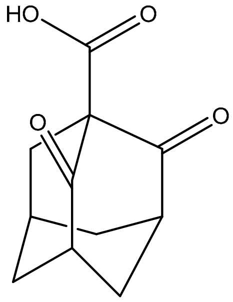
P6	C10H12O2	11.920	163.168	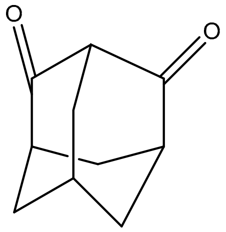

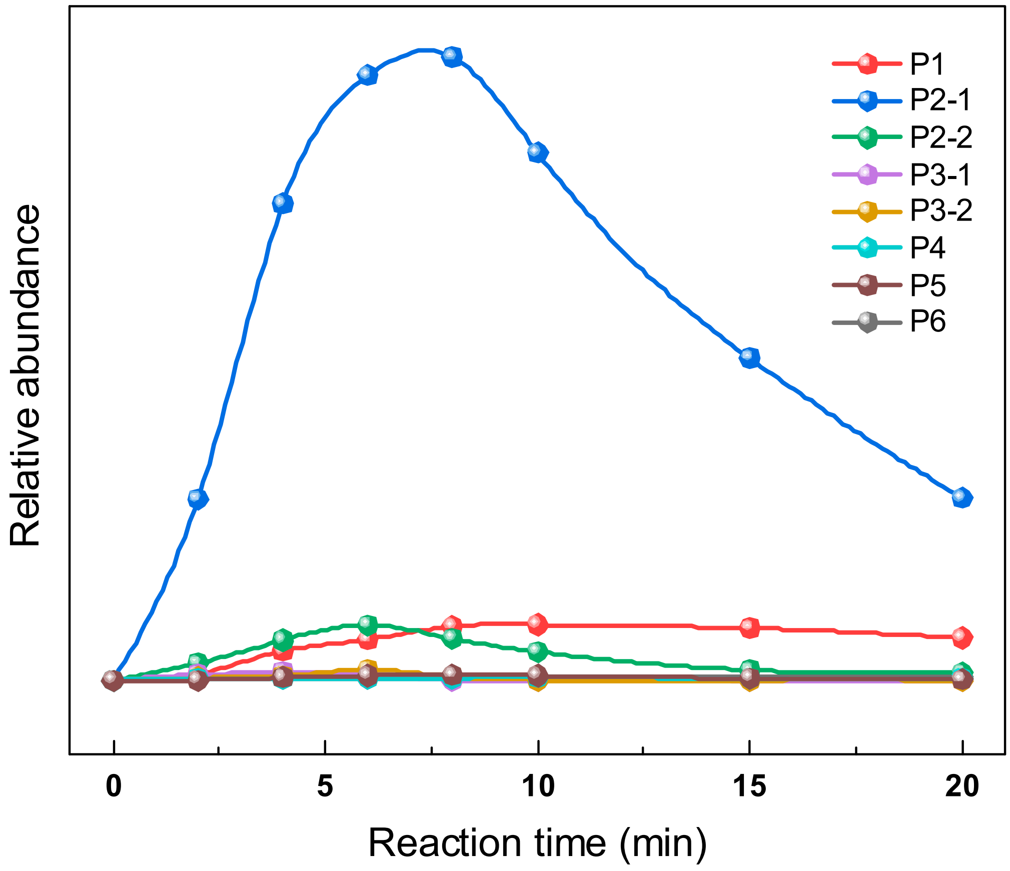

Figure S3. The relative abundance trends of all byproducts generated during the EC/PMS/Fe(III)-EDDS system removing ACA.

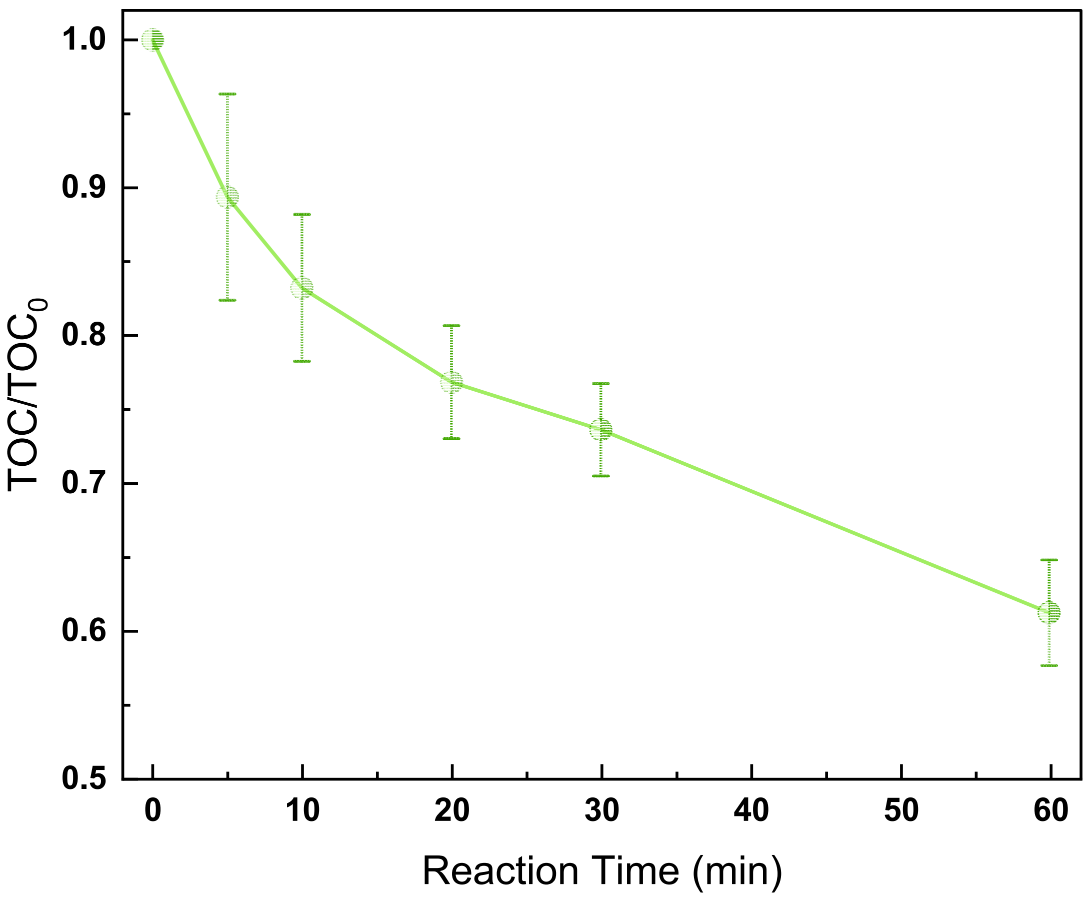

Figure S4. The TOC degradation during the reaction process in the EC/PMS/Fe(III)-EDDS system. Reaction conditions: [ACA] = 0.1 mM, [PMS] = 1 mM, [EDDS] = 0.6 mM, [Fe(III)] = 0.3 mM, [Na_2SO_4] = 20 mM, current density = 8.163 mA/cm^2 and initial pH = 7.0.

Table S3. The distributions of classical NAs, in terms of carbon number and double-bond equivalent (DBE) number, before treatment in the EC/PMS/Fe(III)-EDDS system.

DBE<br>Concentration (mg/L)<br>Carbon Number	0	1	2	3	4	5
**7**	0.04135	0.00757				
**8**	0.30954	0.12481				
**9**	1.44262	0.41762	0.00989			
**10**	2.46248	0.74505	0.04885			
**11**	1.02335	0.66417	0.10598		0.00631	
**12**	0.23794	0.3299	0.13345	0.00641	0.00651	
**13**	0.85536	0.1945	0.15028	0.02413	0.00758	
**14**	0.6538	0.14682	0.17225	0.00483	0.01017	0.00677
**15**	0.69278	0.0607	0.17077	0.0829	0.00455	0.01004
**16**	0.26217	0.05713	0.14211		0.02746	0.0121
**17**	0.01502	0.02931	0.07398	0.05694	0.01957	0.0117
**18**	0.1829	0.01624	0.03939	0.0383		
**19**	1.07447	0.10033	0.02114			
**20**	0.10522					
**21**	0.01					

Table S4. The distributions of classical NAs, in terms of carbon number and double-bond equivalent (DBE) number, after treatment in the EC/PMS/Fe(III)-EDDS system.

Concentration (mg/L) / DBE / Carbon Number	0	1	2	3	4	5
**7**						
**8**	0.01196					
**9**	0.05474	0.01556				
**10**	0.09385	0.02875				
**11**	0.04474	0.02596	0.00418			
**12**	0.01255	0.01309	0.00487			
**13**	0.04502	0.0092	0.00587			
**14**	0.04182					
**15**	0.05514					
**16**	0.17766	0.00918				
**17**	0.01299					
**18**	0.12288	0.00879	0.00827			
**19**	0.52961	0.03552				
**20**	0.04502					
**21**						

Table S5. Chemicals and Reagents

Chemicals and Reagents	Vendor
1-Adamantanecarboxylic acid (ACA)	Adamas Reagent Co., Ltd. (Shanghai, China)
potassium peroxomonosulfate (PMS)	Adamas Reagent Co., Ltd. (Shanghai, China)
$Fe_2(SO_4)_3$	Adamas Reagent Co., Ltd. (Shanghai, China)
tert-butanol (TBA)	Adamas Reagent Co., Ltd. (Shanghai, China)
furfuryl alcohol (FFA)	Adamas Reagent Co., Ltd. (Shanghai, China)
$NaHCO_3$	Adamas Reagent Co., Ltd. (Shanghai, China)
NaCl	Adamas Reagent Co., Ltd. (Shanghai, China)
$NaNO_3$	Adamas Reagent Co., Ltd. (Shanghai, China)
p-hydroxybenzoic acid (p-HBA)	Adamas Reagent Co., Ltd. (Shanghai, China)
coumarin	Adamas Reagent Co., Ltd. (Shanghai, China)
1,3-diphenylisobenzofuran (DPBF)	Adamas Reagent Co., Ltd. (Shanghai, China)
ethylenediamine-N,N'-disuccinic (EDDS)	Aladdin Bio-Chem Technology Co., Ltd. (Shanghai, China)
humic acid (HA)	Aladdin Bio-Chem Technology Co., Ltd. (Shanghai, China)
5,5-dimethyl-1-pyrrolidine-N-oxide (DMPO)	Aladdin Bio-Chem Technology Co., Ltd. (Shanghai, China)
2,2,6,6-tetramethyl-4-piperidinyloxyl (TEMP)	Aladdin Bio-Chem Technology Co., Ltd. (Shanghai, China)
L-histidine	Chengdu Kelong chemical reagent company (Chengdu, China)
methanol	Chengdu Kelong chemical reagent company (Chengdu, China)
Na_2SO_4	Chengdu Kelong chemical reagent company (Chengdu, China)
NaOH	Chengdu Kelong chemical reagent company (Chengdu, China)
H_2SO_4	Chengdu Kelong chemical reagent company (Chengdu, China)
ethanol (EtOH)	Chengdu Kelong chemical reagent company (Chengdu, China)
myristic acid-1-^{13}C	Sigma-Aldrich China
Fluka commercial NAs	Sigma-Aldrich China

Text S2. UHPLC-QTOF-MS analysis of ACA

The ultrahigh performance liquid chromatography quadrupole time-of-flight mass spectrometry (UHPLC-QTOF-MS, G6545, Agilent, USA) with an electrospray ionization (ESI) source and a reverse-phase C18 column (3 μm, 2.1 × 50 mm) (CNW, Germany) was used to characterized ACA. The column temperature was set to 45 °C. The mobile phase was deionized water (A) and pure acetonitrile (B). The flow rate was at 0.4 mL/min, and the injection volume was 2 μL. The instrument was switched to negative ionization and full scan mode. The elution process is divided into three stages: (i) 0-0.2 min, 5% B. (ii) 0.2-3 min, increased from 5% to 95% B (iii) 3-4.5 min, from 95% to 5% B [2,12].

Text S3. UHPLC-QTOF-MS analysis of ACA degradation byproducts

The UHPLC-QTOF-MS with BEH C18 column (100 mm × 2.1 mm, 1.7 μm) (Waters, Milford, USA) was used to identify the byproducts in the degradation of ACA. The column temperature was set to 45 °C. The aqueous phase A was water with 0.1% formic acid (v/v) and organic phase B was pure acetonitrile. The flow rate was aet to 0.1 mL/min. The instrument was set into negative ionization and full scan mode [2,12].

Text S4. UHPLC-QTOF-MS analysis of Fluka commercial NAs mixture

The UHPLC-QTOF-MS with BEH C18 column (100 mm × 2.1 mm, 1.7 μm) (Waters, Milford, USA) was used to analysis the commercial NAs. What is different is that the final sample consisted of 0.5 ml sample, 0.4 ml pure methanol and the last 0.1 milliliter of 4.0 mg/L internal standard (myristic acid-1-^{13}C). The column temperature was set to 45 °C. The mobile phase was divided into the aqueous and organic phase that were water with 2 mM ammonium acetate and a mixture of methanol and acetonitrile (1: 1, v/v) with 2 mM ammonium acetate, respectively. The flow rate was at 0.4 mL/min. The instrument was at negative ionization mode [12,13].

Text S5. The analysis of singlet oxygen ($^1O_2$)

1, 3 - diphenylisobenzofuran (DPBF) as a $^1O_2$ trapping agent was employed to analyze the $^1O_2$ concentration. The concentration of $^1O_2$ was obtained by degraded DPBF due to the molar ratio of them being 1:1. The DPBF concentration was determined by a UV-2600 Shimadzu ultraviolet-visible spectrophotometry (Japan) at the λ = 410 nm.

Text S6. The analysis of hydroxyl radical (•OH)

The high performance liquid chromatography (HPLC) (Prominence RF-20A, Shimadzu, Japan) equipped with a reverse-phase C18 column (4.6 × 150 mm) and a fluorescence (FLR) detector was used to detect the 7-hydroxycoumarin (7-HC) to reflect the content of hydroxyl radicals. It was reported that the coumarin could combines with •OH to form 7-HC. The column temperature was set to 30 °C. The mobile phase was methanol/0.1% ammonium acetate (50:50, v/v). The flow rate was 30 mL/min. The fluorescence intensity was measured using a FLR detector with excitation wavelength of 346 nm and detection wavelength of 456 nm [14].

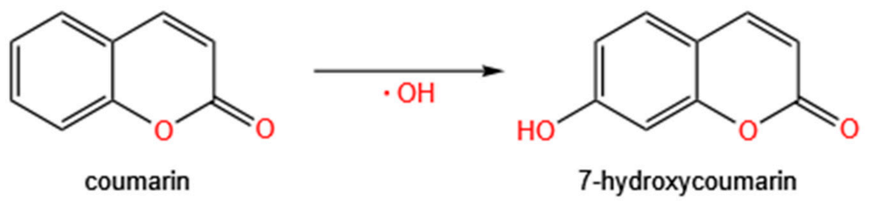

Text S7. The analysis of sulfate radical ($SO_4^{\bullet-}$)

HPLC (1260 Infinity, Agilent, USA) equipped with a DAD detector was used to detect the *p*-hydroxybenzoic acid (*p*-HBA) and *p*-benzoquinone (BQ) to reflect the content of sulfate radicals. It was reported that BQ was the major product of reaction between $SO_4^{\bullet-}$ and p-HBA. The column temperature was set at 30 °C. The isometric elution was carried out with 50% pure water and 50% acetonitrile at a flow rate of 1 mL /min. The absorption wavelength of *p*-HBA and BQ is 246 nm [15].

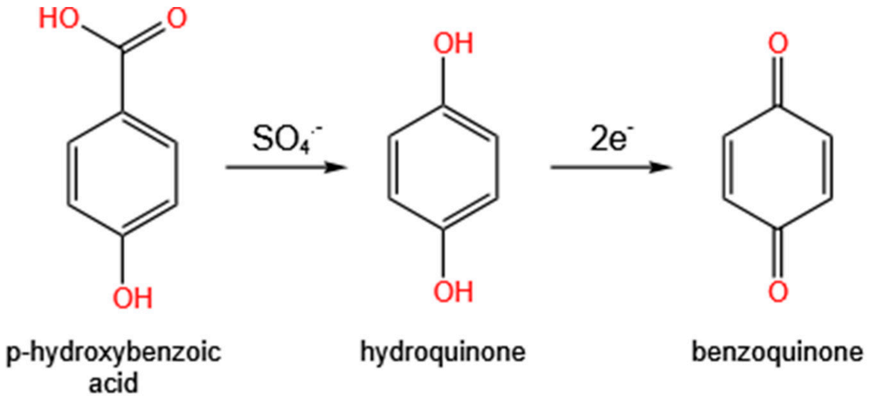

Text S8. The analysis of iron species and PMS

The concentrations of iron species and PMS were determined mainly by chromogenic reaction. The measurement of total dissolved iron and dissolved Fe(II) were measured by UV-vis spectrophotometer (UV-3600, Shimadzu, Japan). Samples were taken at specific time intervals with filtration using 0.22 μm polyether sulfone membranes. For determination of dissolved Fe(II) concentration, 3 mL filtered sample was mixed with 0.1 mL 10 mM 1,10-phenanthroline. For determination of total dissolved iron, 3 mL filtered sample was mixed with 0.1 mL 10 mM 1,10-phenanthroline and 0.1 mL 100 mM of ascorbic acid solution. Noted that the mixed solution should be shaken evenly and measured the absorbance within 20 s, because dissolved Fe(II) was susceptible to oxidization. The absorbance of the mixed solution was detected by a UV-vis spectrometer at 510 nm for measuring the concentrations of total dissolved iron and dissolved Fe(II) using a standard curve method.

The PMS concentration was measured by an UV-vis spectrometer (UV-3600, Shimadzu, Japan). Firstly, 10 mM of 2,2-azino-bis(3-ethylbenzonthiazoline)-6-sulfonic acid diammonium (ABTS) and 10 mM of cobalt sulfate (Co(II)) were prepared before reaction. Sample (1ml) was taken in 10 mL cuvette at specified time intervals and topped to 10 mL with ultrapure water. And then, 0.4 mL 10 mM of ABTS and 0.2 mL 10 mM Co(II) were added. After shaking for 20 min, the absorbance was measured at $\lambda = 735$ nm by an UV-vis spectrometer and the PMS concentration was calculated using a standard curve method [3].

References

1. J. Song, Z. T. How, Z. Huang and M. Gamal El-Din Biochar/iron oxide composite as an efficient peroxymonosulfate catalyst for the degradation of model naphthenic acids compounds. *Chemical Engineering Journal* **2022**, 429, 10.1016/j.cej.2021.132220
2. R. Qin, Z. T. How and M. Gamal El-Din Photodegradation of naphthenic acids induced by natural photosensitizer in oil sands process water. *Water Res* **2019**, 164, 114913. 10.1016/j.watres.2019.114913
3. Y. Chen, Y. Li, Y. Wang, I. Y. Zhang and R. Huang Efficient removal of recalcitrant naphthenic acids with electro-cocatalytic activation of peroxymonosulfate by Fe(III)-nitrilotriacetic acid complex under neutral initial pH condition. *J Hazard Mater* **2023**, 455, 131524. 10.1016/j.jhazmat.2023.131524
4. Y. Zhang, N. Klamerth, P. Chelme-Ayala and M. Gamal El-Din Comparison of Nitrilotriacetic Acid and [S,S]-Ethylenediamine-N,N'-disuccinic Acid in UV-Fenton for the Treatment of Oil Sands Process-Affected Water at Natural pH. *Environ Sci Technol* **2016**, 50, 10535-10544. 10.1021/acs.est.6b03050
5. Z. Luo, L. Meng, Z. T. How, P. Chelme-Ayala, L. Yang, C. Benally and M. Gamal El-Din Treatment of oil sands process water by the ferric citrate under visible light irradiation. *Chemical Engineering Journal* **2022**, 429, 10.1016/j.cej.2021.132419
6. J. Peng, P. Zhou, H. Zhou, W. Liu, H. Zhang, C. Zhou, L. Lai, Z. Ao, S. Su and B. Lai Insights into the Electron-Transfer Mechanism of Permanganate Activation by Graphite for Enhanced Oxidation of Sulfamethoxazole. *Environ. Sci. Technol.* **2021**, 55, 9189-9198. 10.1021/acs.est.1c00020
7. J.-D. Chai and M. Head-Gordon Long-range corrected hybrid density functionals with damped atom-atom dispersion corrections. *Phys. Chem. Chem. Phys.* **2008**, 10, 6615-6620. 10.1039/b810189b
8. A. V. Marenich, C. J. Cramer and D. G. Truhlar Universal Solvation Model Based on Solute Electron Density and on a Continuum Model of the Solvent Defined by the Bulk Dielectric Constant and Atomic Surface Tensions. *J. Phys. Chem. B* **2009**, 113, 6378-6396. 10.1021/jp810292n
9. T. Lu and F. Chen Multiwfn: A multifunctional wavefunction analyzer. *J. Comput. Chem.* **2012**, 33, 580-592. 10.1002/jcc.22885
10. J. Olah, C. Van Alsenoy and A. B. Sannigrahi Condensed fukui functions derived from stockholder charges: Assessment of their performance as local reactivity descriptors. *J. Phys. Chem. A* **2002**, 106, 3885-3890. 10.1021/jp014039h
11. W. Liu, Y. Li, F. Liu, W. Jiang, D. Zhang and J. Liang Visible-light-driven photocatalytic degradation of diclofenac by carbon quantum dots modified porous g-C3N4: Mechanisms, degradation pathway and DFT calculation. *Water Res.* **2019**, 151, 8-19. 10.1016/j.watres.2018.11.084
12. A. S. Abdalrhman, C. Wang, Z. T. How and M. Gamal El-Din Degradation of cyclohexanecarboxylic acid as a model naphthenic acid by the UV/chlorine process: Kinetics and by-products identification. *J Hazard Mater* **2021**, 402, 123476. 10.1016/j.jhazmat.2020.123476
13. Y. Chen, X. Long, R. Huang, I. Y. Zhang, G. Yao, B. Lai and Z. Xiong Highly efficient

electro-cocatalytic Fenton-like reactions for the degradation of recalcitrant naphthenic acids: Exploring reaction mechanisms and environmental implications. *Chemical Engineering Journal* **2022**, 450, 10.1016/j.cej.2022.138331

14. P. Zhou, J. Zhang, G. Zhang, W. Li, Y. Liu, X. Cheng, X. Huo, Y. Liu and Y. Zhang Degradation of dimethyl phthalate by activating peroxymonosulfate using nanoscale zero valent tungsten: Mechanism and degradation pathway. *Chemical Engineering Journal* **2019**, 359, 138-148. 10.1016/j.cej.2018.11.123
15. F. Ji, H. Zhang, X. Wei, Y. Zhang and B. Lai Efficient degradation of atrazine by Co-NZ catalyst prepared by electroless plating in the presence of peroxymonosulfate: Characterization, performance and mechanistic consideration. *Chemical Engineering Journal* **2019**, 359, 1316-1326. 10.1016/j.cej.2018.11.049
